# CT-based radiomics combined with deep learning for predicting radiation pneumonitis in patients with esophageal cancer: a two-center study

**DOI:** 10.3389/fonc.2026.1613933

**Published:** 2026-05-26

**Authors:** Junjie Yang, Yuxiang Zhang, Yu Zhang, Zhaoyi Li, Xinyu Ke, Qingrou Li, Jinchang Wu, Caihong Li, Lansheng Zhang

**Affiliations:** 1 Department of Radiation Oncology, The Second Affiliated Hospital of Xuzhou Medical University, Xuzhou, China; 2 The First Clinical Medical College of Xuzhou Medical University, Xuzhou, China; 3 Department of Radiation Oncology, The Affiliated Suzhou Hospital of Nanjing Medical University, Suzhou, China

**Keywords:** deep learning, esophageal cancer, radiation oncology, radiation pneumonitis, radiomics

## Abstract

**Purpose:**

This study aims to develop a multimodal prediction model combining radiomics and deep learning techniques to assess the risk of radiation pneumonia (RP) in esophageal cancer patients undergoing radiotherapy.

**Methods:**

This retrospective study enrolled esophageal squamous cell carcinoma (ESCC) patients who received conventional fractionated radiotherapy at two hospitals in China between September 2018 and September 2023. The patients were divided into a training cohort (Hospital I, 116 cases) and a validation cohort (Hospital II, 41 cases). The intraclass correlation coefficient (ICC), Least Absolute Shrinkage and Selection Operator (LASSO), and Boruta were used as feature selection methods, while a support vector machine (SVM) was employed for model construction. Six models were constructed based on a combination of clinical features, radiomic features, and deep learning features. Model performance was evaluated using the area under the receiver operating characteristic (ROC) curve (AUC), calibration curve, and decision curve analysis (DCA). Four different machine learning algorithms were used to assess the best model, with the optimal classifier selected.

**Results:**

The hybrid model combining clinical features, radiomics, and deep learning achieved an AUC of 0.902 in the training cohort and 0.857 in the validation cohort, significantly outperforming the single-modality models. Among various machine learning algorithms, the random forest method demonstrated the best performance in external validation with an AUC of 0.859. Based on DCA and calibration curve analysis, the model showed good net clinical benefit and fit.

**Conclusion:**

The multi-modal predictive model, which integrates radiomics and deep learning techniques, effectively predicts the risk of radiation-induced pneumonitis in esophageal cancer patients after radiotherapy. This approach provides a novel pathway for the early identification and prevention of RP.

## Introduction

Esophageal cancer is one of the most common and fatal malignancies worldwide, with a five-year survival rate remaining low in most countries (1). Radiotherapy (RT), as a crucial treatment modality for esophageal cancer, particularly in patients with locally advanced stages, significantly improves survival rates. However, one of the major side effects of RT—radiation-induced pneumonitis (RP)—has a substantial impact on patients ‘health and quality of life (2). The onset of RP is often delayed, with clinical symptoms including dry cough, dyspnea, and chest pain; in severe cases, it can be life-threatening. Therefore, early prediction and management of this condition are essential (3).

Clinical factors and dose-volume histogram (DVH) information are widely used in predicting radiation-induced pneumonitis (RP), but relying solely on these factors has certain limitations. Nomura et al. (2012) investigated predictors of RP in patients undergoing radiotherapy and highlighted that while clinical factors such as weight loss and tumor staging are crucial for RP prediction, the predictive power of individual clinical factors remains limited. Variations in pulmonary recovery capacity and immune response among patients may lead to differing reactions to radiotherapy (4). Sun et al. (2023) identified chronic obstructive pulmonary disease (COPD), pulmonary infection, and dosimetric parameters (e.g., V20) as independent predictors of RP in esophageal squamous cell carcinoma patients. However, these factors exert multifaceted influences and must be considered comprehensively (5). Solely relying on clinical factors and DVH information often overlooks these underlying risk factors, potentially reducing predictive accuracy.

In recent years, radiomics has emerged as a novel technology capable of extracting quantitative features from medical images, offering deeper insights into the biological behavior of tumors (6). Radiomics reveals the complexity of the tumor microenvironment and correlates with clinical outcomes. In esophageal cancer research, radiomic features have been applied to predict tumor response, local recurrence, and survival rates (7). Concurrently, deep learning, a prominent branch of artificial intelligence, has been extensively utilized in medical imaging analysis. By leveraging deep neural networks, researchers can automatically learn effective features from complex data, thereby improving the accuracy of disease detection and prediction (8). The integration of radiomics and deep learning holds promise for further enhancing RP prediction capabilities.

Although existing studies have partially elucidated potential predictors of RP, they often rely on single clinical metrics or imaging features, lacking comprehensive, multimodal analytical frameworks (9). This study aims to develop an integrated model that combines radiomics and deep learning to enhance the prediction of RP in esophageal cancer patients undergoing radiotherapy. By integrating radiomic features with deep learning algorithms, the goal is not only to identify high-risk patients but also to provide clinicians with personalized patient management strategies. This approach will offer a robust theoretical foundation and significant clinical value for optimizing RP prevention strategies.

## Methods

### Study design

This retrospective study aims to evaluate the effectiveness of radiomics and deep learning models in predicting radiation-induced pneumonitis (RP). The study was approved by the hospital’s ethics committee and adhered to the Declaration of Helsinki, Ethics approval number: (2025)102901.

Inclusion criteria were: (1) age between 45 and 85 years; (2) pathologically confirmed esophageal cancer; and (3) locally advanced patients undergoing definitive chemoradiotherapy. Exclusion criteria were: (1) history of esophageal cancer resection; (2) pulmonary infections unrelated to radiotherapy; (3) treatment interruptions exceeding seven days; and (4) follow-up duration of less than three months or incomplete follow-up data. Additional exclusion criteria were: (1) absence of pre-treatment contrast-enhanced chest CT images, poor image quality, or incomplete lung coverage; (2) prior thoracic radiotherapy; (3) incomplete prescription dose or a total dose <50 Gy; and (4) missing dosimetric parameters.

RP diagnosis was determined collaboratively by a radiation oncologist and a radiologist. Diagnosis was based on diffuse interstitial changes confined to (or predominantly within) the radiotherapy field while excluding other potential causes such as infections or checkpoint inhibitor-related pneumonitis (CIP) (10). At both institutions, all patients underwent chest CT before radiotherapy and at 1, 3, and 6 months after treatment to evaluate therapeutic efficacy and potential RP. RP was classified as grade ≥1 according to the Common Terminology Criteria for Adverse Events (CTCAE, version 5.0). Suspected pneumonitis cases were reviewed independently by two experts who analyzed follow-up imaging and clinical records.

### Data collection

The clinical characteristics of 157 ESCC patients are summarized in **Table 1**. Clinical data were collected for each patient, including age, gender, pathological type, staging, and chemoradiotherapy regimen. Dosimetric parameters such as mean lung dose (MLD), V5, V10, V20, V30, V40, gross tumor volume (GTV), and bilateral lung volume were also recorded. Additionally, the occurrence and severity of RP, as well as follow-up imaging at specified time points, were documented.

**Table 1:** Baseline clinical characteristics of esophageal cancer patients in training set and validation set.

Characteristic	Training Set (116)		Z/t/x2	p	Validation Set (41)		Z/t/x2	p
	Non-RP	RP			Non-RP	RP		
Age	73.52(9.6)	73.17(8.7)	0.17	0.86	74.12(7.2)	74.63(6.1)	-0.232	0.818
Gender								
Male	59	22	0.236	0.627	17	11	0.003	0.96
Female	27	8			8	5		
Smoking history								
Yes	62	18	1.52	0.218	21	4	14.272	0.01
No	24	12			4	12		
Lung disease								
NO	75	22	3.127	0.077	23	11	3.725	0.054
Yes	11	8			2	5		
Chemotherapy								
No	11	3	0.163	0.686	7	2	1.368	0.242
Yes	75	27			18	14		
Diabetes								
No	82	27	1.122	0.289	24	16	0.656	0.418
Yes	4	3			1	0		
Immunotherapy								
N	68	15	9.234	0.002	25	12	6.926	0.008
Yes	18	15			0	4		
Tumor site								
Cervical	4	2	1.241	0.473	1	0	1.166	0.761
Upper thoracic	23	8			8	6		
Middle thoracic	40	11			3	3		
Lower thoracic	19	9			13	7		
Hypertension								
No	66	19	2.043	0.153	17	10	0.131	0.717
Yes	20	11			8	6		
Smoking Index	55.83	66.25	-1.823	0.068	19.12	23.97	-1.58	0.207

Patients from the Second Affiliated Hospital of Xuzhou Medical University (Center 1) were used as the training set, while patients from Xuzhou Cancer Hospital (Center 2) served as the validation set.

### CT examination method

All patients underwent routine contrast-enhanced CT scans covering the neck to the abdomen using a SIEMENS CT scanner, performed both before and after nCRT. The imaging protocol included helical scanning mode, HFS positioning, a tube current of 480 mA, a tube voltage of 120 kV, a slice thickness of 5mm, and an in-plane resolution of 0.7363 × 0.7363 mm/pixel. The contrast-enhanced scan protocol was as follows: iodine-based contrast agent at a dose of 1.5 ml/kg, with arterial phase scanning initiated 27 seconds after injection. For this study, esophageal CT arterial phase images with a uniform slice thickness of 5mm were used.

### Radiomics features

#### Image preprocessing

CT images from patients were uniformly processed, starting with the selection of pre-radiotherapy CT scans for radiomics feature extraction. The image processing steps included:

N4 Bias Field Correction: Ensures baseline comparability of images obtained under different imaging protocols.

Image Resampling: Converts images from one pixel grid to another, altering image resolution and dimensions to ensure uniform resolution across images with different original resolutions.

Segmentation and Feature Extraction

A radiologist with three years of diagnostic experience used the MONAIAuto3DSeg plugin within the 3D Slicer platform (3D Slicer image computing platform | 3D Slicer) to automatically segment both lungs. This initial segmentation was refined by a radiation oncologist with 20 years of clinical experience, ensuring exclusion of mediastinal structures such as the esophagus, bronchi, and major pulmonary vessels. Radiomics features of the lungs were then extracted using the open-source PyRadiomics toolbox in Python.

#### Deep learning features

Convolutional Neural Networks (CNNs) are a type of feedforward neural network that excel in processing and analyzing large volumes of images (11). A basic CNN typically consists of three main components: convolutional layers, activation layers, and pooling layers. The output of these layers forms a specific feature space for each image. For task-specific purposes, these feature spaces are connected to fully connected layers to map images to their corresponding labels (12, 13). Therefore, the convolutional layers of CNNs were selected for feature extraction in this study (14).

Different CNN architectures vary significantly in structure. Generally, increasing the depth of a neural network improves its accuracy (15). However, deeper models require learning more parameters and are more prone to issues such as vanishing gradients. Deep learning also demands larger datasets compared to traditional machine learning or probabilistic methods (10). Due to privacy concerns and restrictions on data sharing, medical datasets are often limited in size. Consequently, transfer learning is commonly used in model development (16). Transfer learning involves adopting pre-trained models from other domains and using data augmentation techniques to expand the dataset for fine-tuning the model.

**Figure 1:**
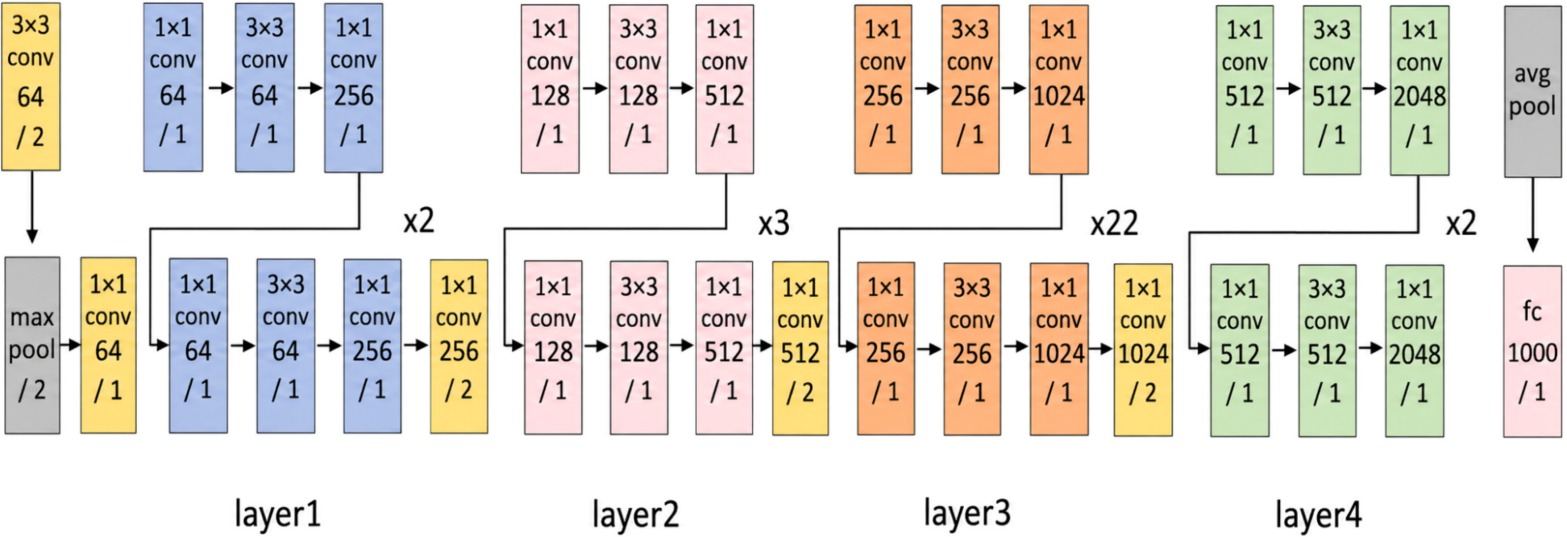
Structure of 3D-Resnet.

ResNet, introduced by researchers from Microsoft Research Asia in 2015, addresses issues such as vanishing gradients, exploding gradients, and accuracy degradation in training deep neural networks (17). Drawing inspiration from the ResNet architecture, we adapted its structure by replacing the 2D convolutional kernels in its convolutional layers with 3D convolutional kernels. Additionally, the output layer of the model was modified to suit the binary classification task specific to this study. This resulted in the development of a 3D-ResNet deep convolutional network model, as illustrated in **Figure 1**.

To retrain the parameters of the 3D-ResNet model, the 116 patients in the training set were divided into RP and non-RP groups. The 3D-ResNet classification model was trained based on this grouping. During training, hyperparameter tuning was conducted using five-fold cross-validation exclusively within the training set. The independent validation cohort was strictly reserved for external evaluation and was not used in any stage of model training or parameter optimization. This approach was adopted to prevent information leakage and ensure the robustness of the model. The final hyperparameter settings for model training were as follows:

Batch size: 8

Epochs: 1000

Optimizer: Adam

Although the number of training epochs was set to 1000, several strategies were employed to mitigate overfitting. Specifically, transfer learning was adopted, and only high-level features extracted from the pretrained 3D-ResNet were used rather than training a deep model from scratch. In addition, a multi-step feature selection approach (ICC, LASSO, and Boruta) was applied to reduce feature redundancy.

### Feature selection and model development

**Figure 2:**
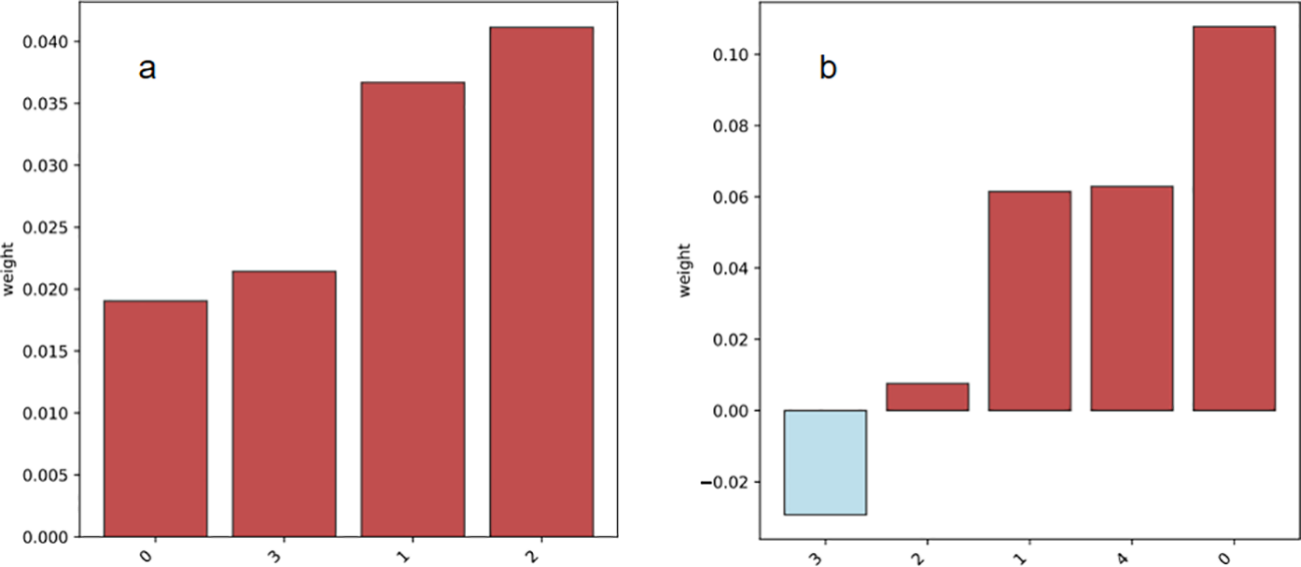
The feature coefficient bar plot, **(A)** Deep learning, **(B)** Radiomics. To construct the RP prediction models, valuable clinical features were selected using independent sample t-tests. After marking the ROIs, 512 radiomics features were extracted via PyRadiomics, and 2048 deep learning features were derived from the ResNet-18 model. To prevent overfitting, intra-class correlation coefficients (ICCs) were calculated for each feature pair, and one of two features with ICC > 0.9 was removed. Feature selection was further refined using LASSO combined with Boruta, resulting in 5 radiomics features and 4 deep learning features.

To mitigate the impact of scale discrepancies among diverse features during model training, all continuous numerical variables (such as V30, MLD, VGTV/Vlung, radiomic features, and deep learning features) were standardized utilizing the z-score method, ensuring a mean of 0 and a standard deviation of 1. Conversely, binary features, such as immunotherapy status, were maintained in their original 0/1 encoding to preserve their categorical significance. (Immunotherapy,V30,MLD,VGTV/Vlung), 5 radiomics features (original_glcm_Correlation,log-sigma-1-mm-3D_glszm_ZoneEntropy,log-sigma-2-mm-3D_gldm_DependenceNonUniformityNormalized,log-sigma-3-mm-3D_firstorder_Maximum,log-sigma-3-mm-3D_glrlm_HighGrayLevelRunEmphasis), and 4 deep learning features (feature 71,feature 86,feature 282,feature 489) being retained. The feature coefficient bar plot is shown in **Figure 2**.

This study used machine learning algorithms like Random Forest and SVM with an RBF kernel to capture nonlinear relationships between clinical features and outcomes. Random Forest aggregates decision trees to model complex interactions, while SVM maps features to a high-dimensional space for optimal separation. Models were trained with consistent data splits and hyperparameters, using five-fold cross-validation and Bayesian optimization for tuning. LassoCV used 10-fold cross-validation repeated three times to find the best regularization parameter, while Bayesian optimization assessed model performance with five-fold cross-validation. The SVM’s regularization parameter C was optimized on a logarithmic scale, and the Random Forest model used out-of-bag sampling and balanced subsample weights to handle class imbalance. All models were trained only on the training set, excluding validation set data. Final performance metrics, including AUC, accuracy, precision, recall, and F1 score, were reported for both training and validation sets to evaluate model generalization.

Six RP prediction models were developed: Clinic, Rad (manual radiomics), Dl (3D-ResNet-18 deep learning), Clinic+Rad, Clinic+Dl, and Clinic+Rad+Dl, with the latter combining all feature modalities for improved performance. We also construct predictive models combining clinical parameters, radiomics, and deep learning features using various classifiers, including SVM(support vector machines), RF(random forests), KNN(k-nearest neighbors), and GD(gradient descent).

### Statistical analysis

Data analysis was conducted using Python (version 3.8.3) and SPSS (version 27.0.1). Differences between variables were assessed using Pearson’s Chi-square test or Student’s t-test, with a significance threshold set at p < 0.05.To build the RP prediction model, the Logistic Regression algorithm was employed. The model’s discriminative ability was evaluated using the Receiver Operating Characteristic (ROC) curve, and performance was measured by calculating the Area Under the Curve (AUC).In addition, the clinical value of the model was assessed by plotting DCA.

## Results

**Figure 3:**
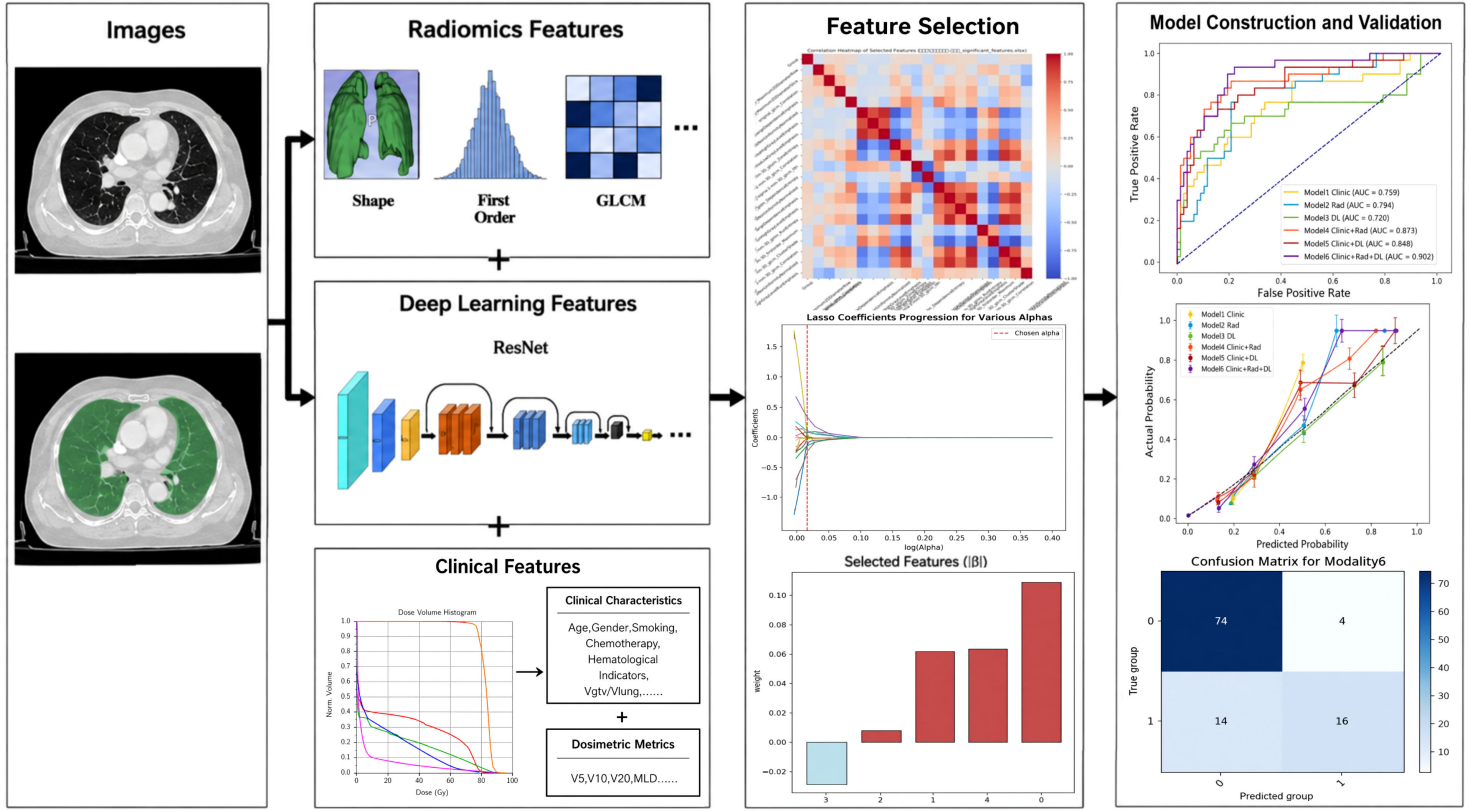
The workflow of the study. Patient Selection. A total of 157 patients with esophageal cancer were enrolled, including 116 patients from the Second Affiliated Hospital of Xuzhou Medical University and 41 patients from Xuzhou Cancer Hospital, between January 2019 and September 2023.

The study flow is depicted in **Figure 3**.The clinical characteristics, hematological parameters and radiation dosimetric characteristics of 157 ESCC patients are summarized in **Table 1**, **Table 2** and **Table 3**. Based on post-treatment chest CT follow-up and subsequent treatments, patients in the training and validation sets were divided into RP groups (30 cases, 16 cases) and non-RP groups (86 cases, 25 cases).Key findings include: Immunotherapy Proportion: The RP group showed a significantly higher proportion of immunotherapy usage compared to the non-RP group in both Center 1 (P=0.002) and Center 2 (P=0.008).Dosimetric Parameters: V30 and MLD values were significantly higher in the RP group than in the non-RP group, with statistical significance.V40 showed no statistically significant difference in the validation set (P=0.276).Tumor Volume Proportion: The ratio of tumor GTV volume to bilateral lung volume was significantly higher in the RP group compared to the non-RP group, with statistical significance in both the training set (P=0.03) and the validation set (P=0.000).

**Table 2:** The hematological parameters of esophageal cancer patients in training set and validation set.

Characteristic	Training Set (116)		Z/t/x2	p	Validation Set (41)		Z/t/x2	p
	Non-RP	RP			Non-RP	RP		
WBC (10^9^/L)	6.03 (2.28)	6.26 (1.95)	-0.5	0.62	6.27 (3.553)	5.83 (2.066)	0.449	0.656
Hb (g/L)	124.24 (17.34)	122.78 (14.9)	0.41	0.68	122.81 (13.49)	125.94 (14.24)	-0.711	0.481
PLT (10^9^/L)	227.92 (64.19)	234.90 (69.41)	-0.5	0.62	204.24 (48.08)	229.88 (69.78)	-1.395	0.171
NEU (109/L)	4.02 (1.96)	4.28 (1.62)	-0.66	0.51	4.48 (3.35)	3.87 (1.92)	0.666	0.509
LYM (10^9^/L)	1.35 (0.53)	1.36 (0.35)	-0.09	0.93	1.11 (0.47)	1.31 (0.44)	-1.392	0.172
ALB (g/L)	39.27 (3.72)	40.48 (3.58)	-1.55	0.12	38.62 (3.48)	38.65 (2.63)	-0.026	0.98
D-D (µg/L)	235.31 (408.34)	230.82 (218.46)	0.06	0.95	229.31 (302.32)	225.31 (253.46)	0.13	0.85
NLR	3.53 (2.3)	3.37 (1.71)	0.33	0.74	4.85 (4.15)	3.30 (1.96)	1.392	0.172

WBC, white blood cell; Hb, Hemoglobin; PLT, Platelet; NEU, neutrophil count; LYM, Lymphocyte; ALB, Albumin; D-D, D-dimer; NLR, neutrophil to lymphocyte ratio.

**Table 3:** The radiation dosimetric parameters of esophageal cancer patients in training set and validation set.

Characteristic	Training Set (116)		Z/t/x2	p	Validation Set (41)		Z/t/x2	p
	Non-RP	RP			Non-RP	RP		
V5	37.24(11.76)	42(12.54)	-1.88	0.06	36.78(12.08)	39.06(13.49)	-0.563	0.577
V10	24.85(9.23)	28.66(9.75)	-1.92	0.06	24.59(9.70)	26.56(10.33)	-0.619	0.539
V15	18.81(8.07)	22.29(8.31)	-2.02	0.05	18.52(8.31)	20.89(8.61)	-0.876	0.387
V20	14.37(6.78)	17.34(7.44)	-2.01	0.05	14.12(6.88)	16.27(7.49)	-0.944	0.351
V25	10.30(5.2)	12.54(6.38)	-1.91	0.06	10.15(5.15)	11.87(6.43)	-0.944	0.351
V30	6.88(4.36)	9.69(5.27)	-2.63	0.01	6.97(4.29)	9.72(5.28)	-2.21	0.01
V40	3.45(2.67)	5.59(3.46)	-3.09	0	3.66(2.76)	4.71(3.33)	-1.104	0.276
MLD	8.32(2.81)	10.77(4.98)	-2.56	0.02	8.89(2.96)	10.20(5.03)	-0.92	0.03
GTV/lung	0.015(0.012)	0.03(0.012)	-3.43	0.03	0.019(0.011)	0.034(0.014)	-3.989	0

**Figure 4:**
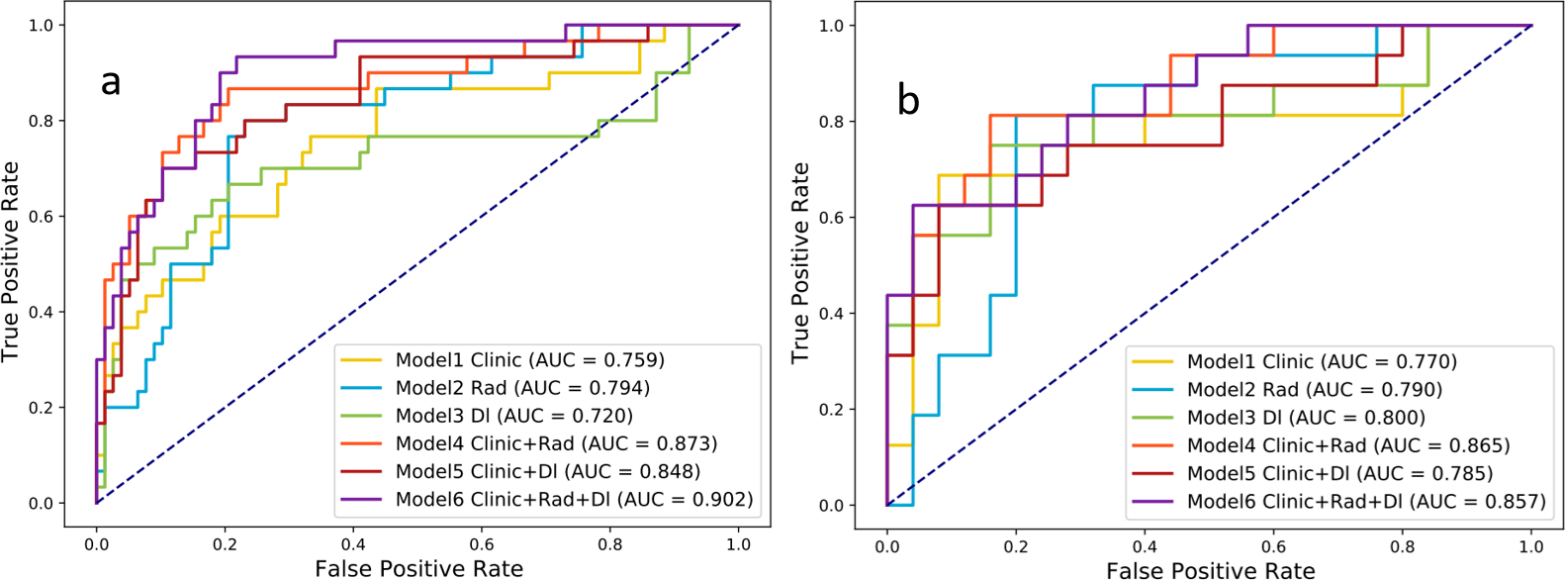
The ROC curve. **(A)** Training set; **(B)** Validation set. AUC, area under the curve.

**Figure 5:**
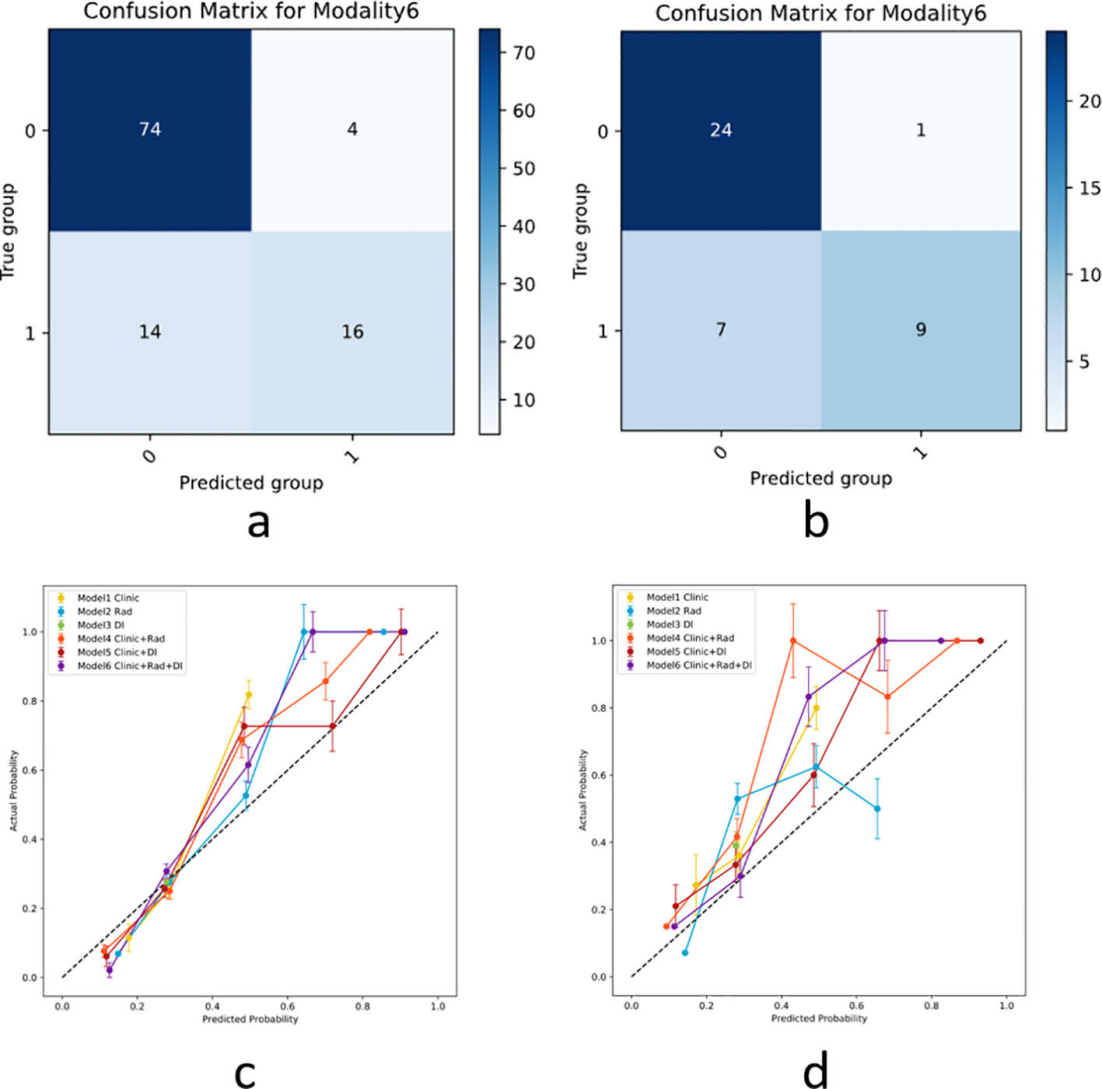
The confusion matrix and calibration curve. **(A)** confusion matrix in training set by model6; **(B)** Confusion matrix in validation set by model6; **(C)** calibration curve in training set; **(D)** calibration curve in validation set.

The Rad model, built with manually extracted radiomics features, showed good performance with AUCs of 0.794 in the training set and 0.790 in the testing set. The Dl model, utilizing deep learning features from 3D-ResNet, achieved AUCs of 0.720 and 0.800 in the training and testing sets, respectively. The combined model integrating clinical, radiomics, and deep learning features significantly improved performance, with AUCs of 0.902 in the training set and 0.857 in the testing set, outperforming both individual and dual-modality models (**Figures 4A, B**). The combined model’s results are further supported by the confusion matrix (**Figures 5A, B**) and calibration curve (**Figures 5C, D**), demonstrating excellent predictive accuracy and calibration.

**Figure 6:**
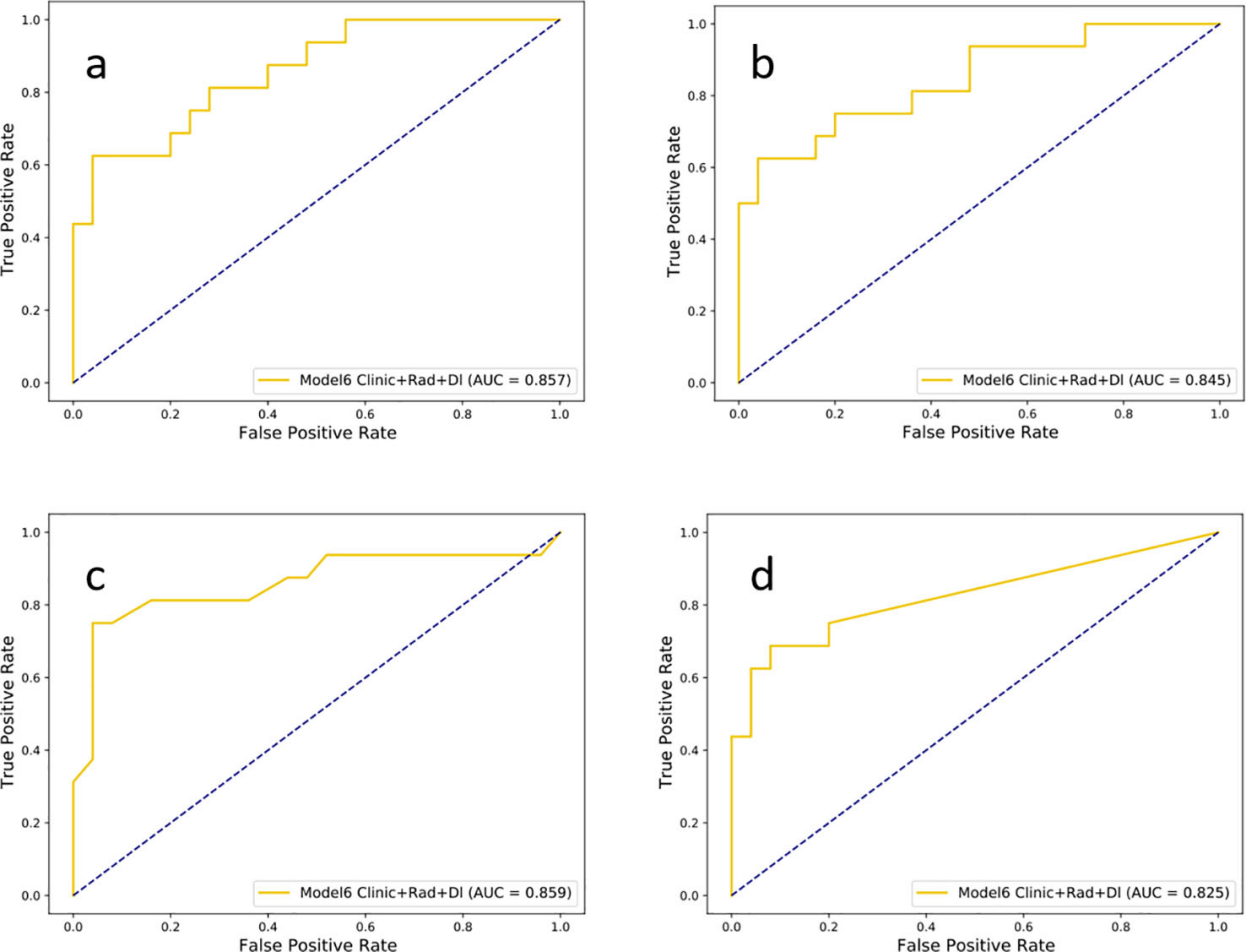
The ROC curves of different classifiers in Model 6. **(A)** SVM; **(B)** GD; **(C)** RF; **(D)** KNN.

**Figure 7:**
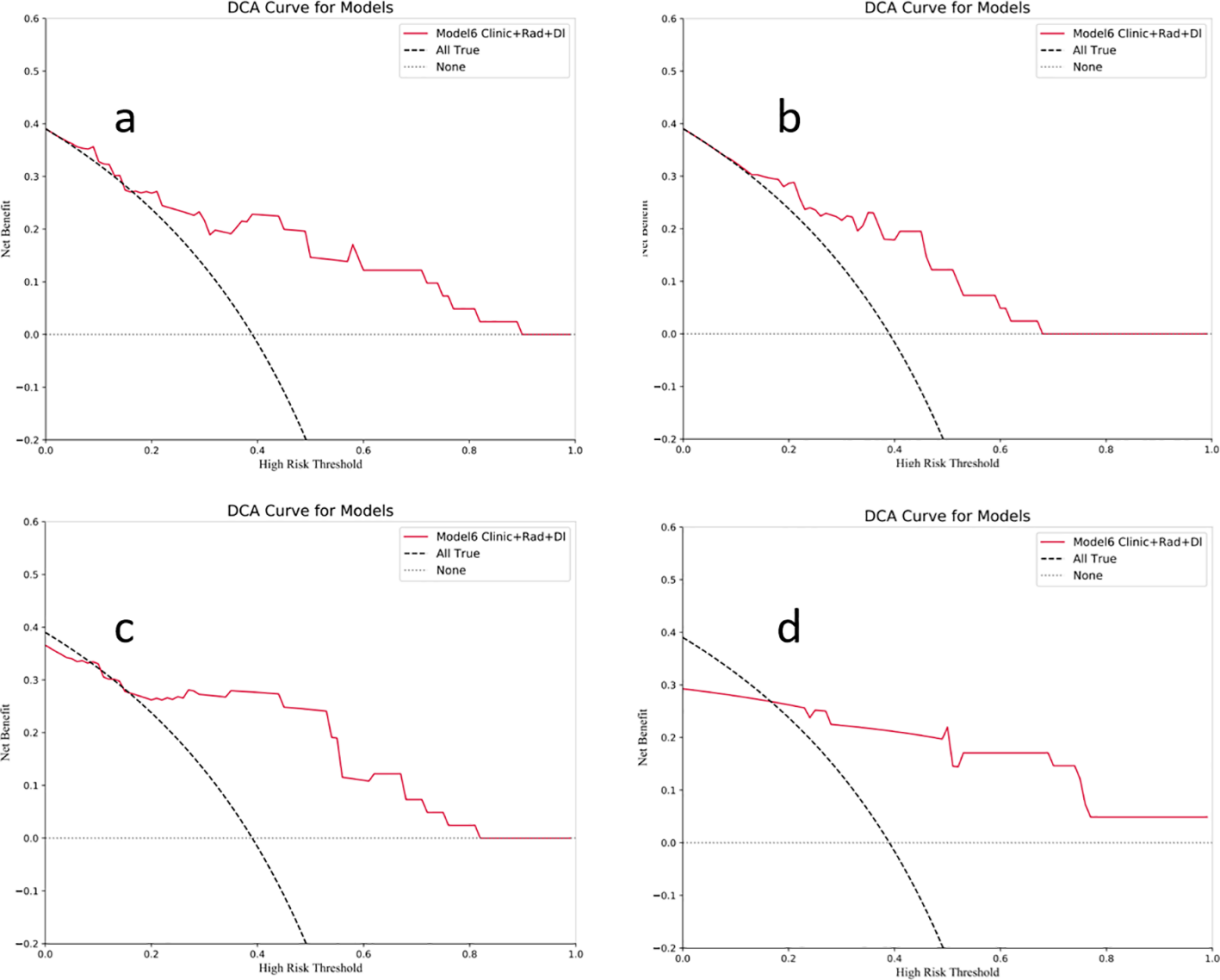
The decision curves of different classifiers in Model 6. The DCA show that the models all have a good net clinical benefit. **(A)** SVM; **(B)** GD; **(C)** RF; **(D)** KNN.

The performance metrics of radiomics signatures of the three machine learning classifiers based on the cut-off value are shown in **Table 4**. Random forests achieve the best performance in external validation among different machine learning methods, with an AUC of 0.859, outperforming support vector machines (AUC=0.857) and gradient descent (AUC=0.825) (**Figure 6**). The DCA curves indicate that the fusion model provides a favorable net clinical benefit across various classifiers, offering valuable guidance for clinicians in formulating treatment strategies (**Figure 7**).

**Table 4:** Performance of RS2 model based on the cut-off value.

Matrix	Training Set				Validation Set			
	SVM	GD	RF	KNN	SVM	GD	RF	KNN
AUC	0.902	0.895	0.908	0.867	0.857	0.845	0.859	0.825
F1 Score	0.64	0.48	0.88	0.8	0.69	0.47	0.79	0.74
Accuracy	0.83	0.81	1	1	0.8	0.73	0.85	0.83
Recall	0.53	0.33	0.8	0.71	0.56	0.31	0.69	0.63
Precision	0.8	0.91	1	0.92	0.9	1	0.92	0.9
PPV	0.94	0.98	1	1	0.96	1	0.96	0.96
NPV	0.53	0.33	0.8	0.63	0.56	0.31	0.5	0.6

AUC, area under the curve; PPV, positive predictive value; NPV, negative predictive value; SVM, support vector machine; GD, Gradient Descent; RF, Random Forest; KNN, K-Nearest Neighbors.

## Discussion

In this study, we developed and validated a multimodal predictive model integrating clinical, radiomics, and deep learning features for the prediction of radiation pneumonitis (RP) in patients with esophageal cancer undergoing radiotherapy. Our findings underscore the importance of predictive models integrating radiomics features and clinical factors for accurately identifying high-risk patients. Previous research has highlighted key RP risk factors, such as radiation dose, lung tissue volume exposed, and baseline lung function (18). By incorporating radiomics features and deep learning features, our approach captures micro-level changes beyond traditional clinical assessments, improving prediction accuracy. The results of this study indicate that the proportion of immunotherapy usage was significantly higher in the RP group compared to the non-RP group. Additionally, the RP group had higher V30 and MLD values, and the ratio of tumor GTV volume to total lung volume was also greater. Both the radiomics (Rad) model and deep learning (DL) model performed well, with AUCs of 0.794 and 0.720, respectively. The integrated model (which combines clinical, radiomic, and deep learning features) achieved AUCs of 0.902 and 0.857 in the training and testing sets, significantly outperforming the individual models. In external validation, the random forest model showed the best performance (AUC = 0.859). The decision curve analysis (DCA) further demonstrated that the integrated model provided excellent net clinical benefit across different classifiers, offering effective guidance for clinical treatment strategies.

In recent years, deep learning techniques have been increasingly applied in medical imaging analysis. Studies have shown that deep learning models can autonomously extract disease-related features from large imaging datasets, enhancing predictive accuracy and reliability (19) (20). In our study, convolutional neural networks (CNNs) proved effective in extracting relevant features from complex imaging data, aligning with conclusions from similar research (8). For example, Li et al. (2024) enhance the early diagnosis and treatment of esophageal cancer, suggesting that deep learning could serve as a useful tool for esophageal cancer screening (21). Similarly, our findings support the application of deep learning in predicting esophageal cancer-related complications.

Radiomics, as an emerging technology, extracts numerous quantitative features from medical images, offering new opportunities for personalized treatment. Aerts et al. (2014) highlighted that radiomics features reveal tumor biology and correlate with treatment response and prognosis (7). Additionally, Kraus et al. showed that combining dosiomics and radiomics significantly improves RP prediction for patients undergoing stereotactic body radiotherapy (SBRT), enabling personalized treatment planning and reducing side effects (22).

Sheen et al. systematically evaluated various radiomics models for RP prediction, emphasizing that integrating machine learning algorithms with radiomics features enhances predictive accuracy (23). Their study highlighted the value of extracting diverse imaging features, such as texture and morphology, for better prediction across patient groups. Moreover, combining radiomics with dose distribution data helps assess the impact of radiation on lung tissue and optimize treatment plans. In our study, the extracted radiomics features further confirmed their effectiveness in RP prediction, providing a comprehensive perspective for assessing treatment response and potential complications in clinical practice.

Deep learning plays a crucial role in predicting radiation pneumonitis (RP) by automatically extracting complex features from medical images, significantly improving the accuracy of risk prediction. Research has shown that deep learning models, such as convolutional neural networks (CNNs), can identify patterns that traditional methods fail to capture, thereby enhancing the prediction of radiation therapy-related complications (24). Fourcade et al. (2019) demonstrated that deep learning achieves expert-level performance in various medical imaging tasks, laying a strong foundation for its potential in the field (25).Additionally, Kapoor et al. (2023) utilized 3D CNNs to predict RP in patients undergoing stereotactic body radiotherapy (SBRT) by analyzing their three-dimensional dose distribution data, offering a novel automated prediction tool for clinical use (26). Thus, deep learning not only enhances prediction efficiency but also serves as a vital approach to assessing RP risk.

The application of immune checkpoint inhibitors (ICIs) in esophageal cancer treatment has achieved remarkable progress. However, while the combination of ICIs with radiotherapy (RT) demonstrates synergistic antitumor effects, it also poses increased risks of treatment-related side effects, particularly radiation pneumonitis (RP) and immune-related pneumonitis (IRP). These side effects can not only impact the quality of life but also disrupt the treatment process. Radiation-induced RP results from direct damage to normal lung tissues and subsequent inflammatory responses. After radiation exposure, alveolar epithelial and endothelial cells are injured, leading to the release of inflammatory cytokines such as IL-6 and TGF-beta1, which trigger acute inflammation and may progress to pulmonary fibrosis (27). On the other hand, ICIs enhance T-cell-mediated tumor killing by blocking the PD-1/PD-L1 or CTLA-4 pathway (28). However, this immune activation may also target normal lung tissues, causing IRP. When RT and ICIs are combined, their inflammatory mechanisms can overlap. Radiation releases tumor antigens, amplifying the effects of immunotherapy but also activating additional immune cells, thereby increasing the risk of normal tissue damage (29). In the PACIFIC trial, patients with locally advanced, unresectable NSCLC underwent sequential chemoradiotherapy followed by treatment with either durvalumab or a placebo. Pneumonia occurred in 34% of patients receiving durvalumab, compared to 25% in the placebo group (30). Abe et al. (2023) found that combining the immune checkpoint inhibitor durvalumab with chemoradiotherapy improves therapeutic outcomes but significantly increases the risk of immune-related pneumonitis (31). This prospective study highlighted that enhanced immune responses from combined therapy can lead to increased pulmonary toxicity. Similarly, Cui et al. (2023) observed higher risks of RP and immune-related side effects in patients undergoing combined RT and ICIs, particularly affecting lung function during treatment (32). Factors contributing to these risks include RT dose, irradiated volume, type of immunotherapy, and preexisting conditions like chronic obstructive pulmonary disease (COPD) or pulmonary fibrosis.

This study has several limitations. First, although the grading of RP was determined based on a consensus among multidisciplinary clinicians, the retrospective nature of the diagnosis introduces uncertainty and potential bias. Therefore, prospective validation is required. Second, this study utilized a large number of radiomic features to construct the predictive model, which may increase the risk of overfitting. Although deep learning models are typically data-intensive and the sample size in this study is relatively limited, several strategies were implemented to reduce the risk of overfitting. First, transfer learning was employed to leverage pretrained model representations rather than training from scratch. Second, a multi-step feature selection process (ICC, LASSO, and Boruta) was used to reduce feature dimensionality and redundancy. Third, and most importantly, an independent external validation cohort from another center was used, and the model maintained stable performance, supporting its generalizability. However, the current study included only a limited set of clinical features. Future research could incorporate additional RP-related clinical variables, such as pulmonary function metrics, nutritional status, and novel dosimetric parameters, to evaluate whether predictive accuracy can be further improved. In this study, RP grade ≥1 was used as the endpoint to increase sensitivity for early detection of pulmonary changes. However, future studies focusing on clinically significant RP (grade ≥2) are warranted to further enhance clinical applicability.

The introduction of immunotherapy in the treatment of esophageal cancer has demonstrated significant therapeutic benefits. However, when combined with radiotherapy, the overlapping toxicities present new challenges, particularly the occurrence of pneumonia. Our next research plan involves including esophageal cancer patients undergoing combined radiotherapy and immunotherapy to study the prediction of pneumonia induced by the combination therapy. Additionally, we aim to explore the effects of treatment modalities, the sequence of radiotherapy and immunotherapy, and the interval between treatments on therapeutic outcomes and treatment-related adverse events from an imaging perspective.

## Conclusion

This study demonstrates that integrating radiomics features with deep learning models enhances the ability to predict the risk of radiation pneumonitis in patients with esophageal cancer, thereby facilitating personalized clinical management. As technology continues to evolve, this approach holds promise for clinical application, offering new perspectives on patient risk assessment and monitoring.

## Data Availability

The original contributions presented in the study are included in the article/supplementary material. Further inquiries can be directed to the corresponding authors.
